# An asset-based participatory community analysis of natural hazards in Naphuno, Greater Tzaneen Municipality, Limpopo province, South Africa

**DOI:** 10.4102/jamba.v12i1.939

**Published:** 2020-12-14

**Authors:** Allucia L. Shokane, Hanna Nel

**Affiliations:** 1 Department of Social Work, Faculty of Arts, University of Zululand, Richardsbay, South Africa; 2 Department of Social Work, Faculty of Humanities, University of Johannesburg, Johannesburg, South Africa

**Keywords:** natural hazards, community disaster, disaster planning, asset-based community development (ABCD), social work

## Abstract

Natural hazards disrupt the daily lives of people and communities. Consequently, social workers, like any other stakeholders, deal with community predicaments arising from the effects of natural hazards. The social relief distress (SRD) programme of government utilises needs-based, top-down government-driven interventions in communities affected by natural hazards, focused on what communities lack, as opposed to what communities have. This research study involved a community that experienced natural hazards, such as flooding, hail, lightning and windstorms, which destroyed property and livelihoods during the period 2014–2015. Eight experts and 12 affected community members participated in a qualitative participatory action research analysis study between 2016 and 2017. Guided by the asset-based community development (ABCD) approach, the affected community participated in a collaborative manner in the analysis of the consequences of natural hazards within the community. Data were collected through semi-structured individual interviews and focus group discussions, and analysed thematically. The findings confirmed the traumatic effects of natural hazards, such as loss of property, crops and livestock, physical injuries and even death. The main finding established that natural hazards should be managed in a collaborative way between formal experts of natural hazards and community members through ABCD principles and methods in building resilient communities.

## Introduction

The occurrence of natural hazards is escalating in many parts of the world (Collins 2009; Rinkel & Powers 2017; Sarma, Das & Bera 2020). In 2019, 396 natural hazards were recorded, with 11 755 deaths, 95 million people affected and economic losses of $103 billion across the world. The Centre for Research on the Epidemiology of Disasters (CRED 2019) confirmed that a wide range of natural hazards in the world were in the form of floods in India because of the high monsoon rains, which lasted from July to October 2019 and affected 13 states (mainly in North India). Furthermore, the CRED (2019) in the Emergency Events Database (EM-DAT) recorded the deadliest natural hazards events in 2019 in the form of summer heat waves that affected Europe, more specifically France, Belgium and the Netherlands, with over 2500 deaths. Even the African continent was not spared from droughts and storms in 2019 that were deadliest, such as cyclone Idai, which affected central Mozambique and Zimbabwe with over 1200 deaths and some people missing (Centre for Research on the Epidemiology of Disasters [CRED] 2019; Sarma et al. 2020).

Natural hazards are unavoidable; they disrupt the daily lives of people and communities, can result in substantial loss of life and cause social upheaval (Shokane 2017; Zakour & Gillespie 2017). In addition, Sarma et al. (2020) concurred that disasters are unpredictable occurrences of a series of disruptions within a period, which cease the functioning of a society, with potential great loss of human lives and property, as well as impacting the environment. Consequently, all countries should deal with natural hazards at some stage in their developmental process. The Sendai Framework for Disaster Risk Reduction (2015) confirmed that a disaster results from the combination of a hazard, vulnerability and insufficient capacity or measures to reduce the potential changes of risk and often impacts the vulnerable population, causing damage, casualties and disruption which lead to many people becoming homeless, helpless and hungry. Profoundly, poor people are most vulnerable to the effects of natural hazards (Shokane 2016; UNISDR 2015). Consequently, this research study was conducted in the context of community disaster interventions by researchers working with vulnerable populations and from a social work perspective. Mostly, social work intervenes ‘on vulnerable populations as the profession’s core clients’ (Zakour & Gillespie 2017:12). Moreover, social workers have a unique responsibility amongst the ‘helping professionals’ in that they have the additional responsibility of advocating for social justice and human rights on a macro level, particularly on behalf of more vulnerable populations. Furthermore, the Global Agenda for Social Work and Social Development (Global Agenda) endorsed the commitment to action of the social work and social development communities to work towards promoting sustainable communities and environmentally sensitive development (Jones & Truell 2012; Lombard & Twikirize 2018).

As natural hazards affect all populations with intense disruption of the social, economic and environmental conditions necessary for the well-being of people, social workers are therefore expected to intervene ‘in systems at all levels’ (Zakour & Gillespie 2017:7). However, it was earlier argued by Marlow and Van Rooyen (2001:242) that in social work literature ‘only a few authors have taken on the challenge of considering environmental issues and their relationship to social work’. Further research conducted by Dominelli (2012) and Zapf (2009) with Rinkel and Powers (2017) regarded social work after natural hazards as an essential but underdeveloped area of professional scholarship and practice. The reviewed literature confirmed that social workers are dealing with natural hazards but mainly at international level, whereby they participated in various disaster relief efforts, such as after the Asian earthquake and the Indian Ocean Tsunami in 2004, which heavily affected a number of countries (Bliss & Meehan 2008; Fahrudin 2012; Mathbor 2007; Tan 2009; Tang & Cheung 2005; Yanay & Benjamin 2007). This has resulted in worldwide international organisations making a commitment to address disaster issues (Collins 2009; Fahrudin 2012). The UNISDR (2015) observed that during the Tsunami disaster, all international organisations cooperated at all levels to reduce the disaster risk. Furthermore, Bliss and Meehan (2008) demonstrated that social work involvement in disaster response efforts can also be organisational in nature. Significantly, the literature further confirmed that natural hazards occur in different parts of the world and many social work professionals are engaged in the disaster relief process, but with only a few documented in a South African context. The only observation of emerging natural hazard research in South Africa focusing on community social work and natural hazards by environmental-social work scholars such as Shokane and Nel (2017), as well as Rinkel and Powers (2017), with Masoga and Shokane (2019), Lombard and Twikirize (2018) and Shokane (2017, 2017, 2019), ‘have the potential to contribute to an environmentally effective social work practice’ (Marlow & Van Rooyen 2001:252). Thus, social work disaster management and community intervention approaches should tackle the impact of disasters, and seek to make individuals, communities and societies more resilient and less vulnerable to shocks and stresses (Davies et al. 2008; Tanner & Mitchell 2008; Zakour & Gillespie 2017). In South Africa, the Limpopo, Mpumalanga, Gauteng and Northwest provinces experienced heavy rains and floods during late February and early March 2014, which caused a loss of life and extensive damage to property (Gibbs 2014). This retrospective research involved a community that experienced natural hazards, such as flooding, hail, lightning and windstorms, which destroyed property and livelihoods in Naphuno villages of Greater Tzaneen in the Limpopo province.

The gap that this study aims to fill in the existing knowledge is that most natural community disaster intervention efforts in communities affected by natural hazards utilised a needs-based approach, which typically focused on what communities lacked as opposed to what they have. It is thus essential to incorporate asset-based community development (ABCD) interventions in natural disaster management. Asset-based community development is conceptualised by Schenck, Nel and Louw (2010:62) as ‘a people-centred participatory approach to development, based on principles of empowerment and ownership’. The ABCD approach was previously applied in similar studies by Shokane and Nel (2017) whereby they developed community development intervention guidelines for communities affected by natural hazards, and in a doctoral study by Shokane (2017) whereby a participatory community development practice model for rural communities affected by natural hazards was developed.

This study strives to provide guidelines on how to intervene in the case of a community natural hazard utilising community assets of the people affected by natural hazards. Stakeholders who are at the forefront of communities should deal with the impacts of disasters in a collaborative manner. In the context of this study, stakeholders could be social workers, ward councillors, traditional leaders or researchers who specialise in the field of community development, and who ‘are usually “doers” interested in improving their practice through innovation, change and development’ (Zuber-Skerrit & Fletcher 2007:414). The argument raised in this study is that community disaster officials and practitioners should execute the task of intervening in natural hazards through community development, in collaboration with the community concerned utilising their assets. This is significant because community development is primarily about helping people learn how to help themselves (Schenck et al. 2010; Weyers 2011). It is envisaged that the outcome of this study will contribute to all sectors working in the field of disasters to analyse natural hazard challenges, develop intervention plans using community assets within the affected communities and guide any disaster intervention efforts in a collaborative manner. In this article, a theoretical framework applied will be presented. In addition, the research methodology, results and discussion of findings and conclusions and recommendations of the study will be presented.

## Asset-based community development

To plan appropriately for an asset-based community disaster intervention, an ABCD approach pioneered by Kretzmann and McKnight (1993) was selected as an overarching theoretical framework to guide the asset-based angle of this research study. The ABCD approach was also selected for its participatory element of community involvement, and the empowerment of community members affected by natural hazards on how they can better address natural hazards in a participatory way. The ABCD approach was also suitable for the study because it identifies not only problems but also assets and resources present in the community. Furthermore, the ABCD approach was used in this research because of its ability to enable a community to recognise its strengths by understanding what it has, rather than what it needs or lacks (Ennis & West 2010).

The ABCD approach guided the analysis process by assisting participants to develop an inventory of community strengths which contribute to community development and community building (Kettner, Moroney & Martin 2017). In addition, Schenck et al. (2010) maintained that the ABCD approach draws on the resources, abilities and insights of community members to find ways of overcoming their own challenges. In this regard, the ABCD approach was also selected for this research study to assist community members affected by natural hazards to map assets, and to follow this with a planning phase. Furthermore, the ABCD approach focuses on how communities could become self-sustaining by using what they have – that is, the resources of the people and the place – rather than focusing on their needs (Kretzmann & McKnight 1993; Mathie & Cunningham 2009; Schenck et al. 2010). The intention of the researchers was to use the ABCD approach to create a disaster-resilient community, empowering community members affected by natural hazards (Nel 2015; Pretorius & Nel 2012).

## Research methodology

The research methodology selected for this study was participatory action research (PAR), conducted in a qualitative nature. Participatory action research allowed community members affected by natural hazards to participate in the community analysis and planning activities in a collaborative manner (Strydom 2011). The PAR was incorporated with the ABCD approach to guide community members to anticipate disasters and to protect community livelihoods and their assets. The assets could be their homes and possessions, cultural heritage and economic capital. The ABCD approach enhances collaboration between community members and different stakeholders, such as government officials, in becoming disaster-resilient communities (Nicholas, Rautenbach & Maistry 2010). It was therefore appropriate to conduct an analysis of the natural hazard using an asset-oriented approach to community development with the affected community. The assets and strengths of community members were used to create consciousness of how community members may possibly contribute to rebuilding their community after they had experienced a natural hazard, as well as how to act proactively following natural hazards in the future.

### Population and sample

The population of this study was purposively selected from the Naphuno community, which had been affected by natural hazards, such as flooding, hail, lightning and windstorms, that destroyed property and livelihoods during the period 2014–2015 in the Greater Tzaneen Municipality in the Limpopo province of South Africa. The selection of the population was based on the prior knowledge of the researcher as a practising social worker at the Department of Social Development through a number of social relief of distress (SRD) assessments conducted after the community experienced natural hazards. Therefore, the community members affected by natural hazards participated in a participatory analysis from 2016 to 2017, based on how they can incorporate their assets (experiences, skills and assets) in the intervention of future community disasters.

Cooperation and permission by the community were obtained before conducting this study. Initially, permission was sought from the traditional authority as a requirement for entry into many rural villages. The traditional authority acts as a gatekeeper to the community when outsiders wish to gain entry and cooperate with the community. In total, 20 participants were selected for the study, comprising eight experts in community disasters, because of their closeness to the research topic and expertise in the field of community disasters, and 12 participants representing community members from households that have been directly affected by natural hazards (see Table 1) who were selected from a population of 60 community members. Only 20 participants were selected because they were willing and available to participate in the study.

**TABLE 1:** The demographic information of the respondents identified as experts in community disasters (*N* = 20).

Category of respondents	Gender		Age				Language		Justification for inclusion in the study
	M	F	30–40	40–50	50–60	60–70	Sepedi	Xitsonga	
1. Ward councillor A	✓	-	-	✓	-	-	Sepedi	-	Community leaders, who are closely involved in the case of disasters, assist the community by providing them with available resources, such as financial, material and food parcels.
2. Ward councillor B	-	✓	-	✓	-	-	✓	-	
3. Religious leader	✓	-	-	-	✓	-	✓	-	Provide accommodation and shelter in the church for people affected by natural hazards; they also provide traditional and spiritual services for people affected by natural hazards.
4. Traditional leader	✓	-	-	-	✓	-	✓	-	
5. Municipality disaster official	-	✓	✓	-	-	-	✓	-	Because of their professional involvement with disaster management in the community, they work with the ward councillors to provide temporary shelter in the form of tents provided by the Greater Tzaneen Municipality.
6. DSD social worker	✓	-	✓	-	-	-	✓	-	Carry out professional assessment and counselling of disasters and provided a temporary.
7. DSD social worker	-	✓	✓	-	-	-	✓	-	Social Relief of Distress programme for people who had experienced natural hazards.
8. SASSA official	-	✓	-	✓	-	-	✓	-	Process the SRD in terms of social assistance grants through the *Social Assistance Act*, which makes provision of SRD programme after the recommendation of a social worker for a period of 6 months.
	-	-	-	-	-	-	-	-	
9. Community member 1 from household affected by natural hazard	✓	-	-	-	✓	-	✓	-	House roof blown away because of thunderstorm.
10. Community member 2	-	✓	-	✓	-	-	✓	-	Experienced flooding and house built with mud was demolished.
11. Community member 3	✓	-	-	✓	-	-	-	✓	House roof blown away because of thunderstorm.
12. Community member 4	-	✓	-	-	✓	-	✓	-	Lost a family member because of lightning.
13. Community member 5	-	✓	-	-		✓	✓	-	House roof blown away because of thunderstorm.
14. Community member 6	✓	-	-	-	✓	-	✓	-	Family sustained injuries when house collapsed.
15. Community member 7	✓	-	-	✓	-	-	-	✓	House was destroyed by flooding.
16. Community member 8	-	✓	-	-	✓		✓	-	House was destroyed by hailstorm.
17. Community member 9	-	✓	-	-	-	✓	✓	-	Experienced flooding and house built with mud has collapsed.
18. Community member 10	-	✓	-	✓	-	-	✓	-	House roof blown away because of thunderstorm.
19. Community member 11	✓	-	-	-	-	✓	✓	-	House roof blown away because of thunderstorm.
20. Community member 12	✓	-	-	-	✓	-	✓	-	Experienced flooding and house built with mud was demolished.
Total	-	-	-	-	-	-	17	3	Respondents identified experts in community disasters (8).
									Community members affected by natural hazards (12).
**Total**	**10**	**-**	**3**	**7**	**7**	**3**	**17**	**3**	**-**

DSD, Department of Social Development; SASSA, South African Social Security Agency; SRD, social relief distress; M, male; F, female.

Note: The tick signposts the demographic details of the participants, such as gender whether male or female, and the age group whether the participants falls between the age groups of 30–40, 40–50, 50–60 or 60–70 or the Language spoken by respondent whether Sepedi or Xitsonga.

### Data collection

Written informed consent was obtained from all the participants before the commencement of data collection. Semi-structured individual interviews were conducted with the eight experts as indicated in Table 1 that lasted for 1 h each and focus group discussions (FGDs) set up with the 12 community members for six sessions of 2 h per session. The community members were directly affected by natural hazards during the period 2014–2015 (see Table 1 for more clarity). The main study research questions were formulated to analyse the effects of natural hazards in the community; how to establish participatory community strategies that can be used to manage natural hazards and how a community affected by natural hazards can rebuild its livelihood after a disaster. The purpose of the interviews and FGD was to analyse the problems caused by natural hazards, and the opportunities to rectify the problems created. This step was essential as it recognises that participants are capable of analysing their own situation and developing opportunities or solutions for the challenges they face (eds. De Vos et al. 2011; Vollman, Anderson & McFarlane 2004).

Storytelling as an appreciative inquiry (AI) method was used in the FGD to allow community members to share their experiences of natural hazards in terms of fears, problems, solutions and opportunities. Solution-focused discussions enhance participants’ agency and motivation which lead to self-reliance (Mathie & Cunningham 2009). The AI theory was developed by Cooperrider and Srivasta in the 1980s and is based on the premise that ‘organisations change in the direction which they enquire’ (Myers 1994:173). Myers (1993) and Schenck et al. (2010) affirmed that a community that ‘enquires’ into problems will keep finding problems, whereas a community that appreciates what it is best at will discover more of what it is good at. During the FGD, the participants were asked to conduct a capacity skills inventory to identify the community’s ‘individual gifts, skills and capacities’ (Schenck et al. 2010:162). This ‘head, hand and heart’ tool was used to analyse the capabilities, talents and skills of community members (Mathie & Cunningham 2009). Although the emphasis of this phase focused on problems of natural hazards, positive aspects in line with the ABCD approach were integrated into the problem analysis phase. Furthermore, the tradition of AI, which is at the root of ABCD, supported the identification of some of these skills and attributes (Ashford & Patkar 2001; Schenk et al. 2010).

It is worth noting that the type of community intervention-based research conducted in this study was a complex and multifaceted process that requires a long-term effort ‘consisting of a series of distinct but interconnected phases performed over time’ (eds. Rothman & Thomas 1994:69). Remarkably, the researcher had a prolonged engagement in the research and had stayed in the field from February 2015 to January 2016 for a period of 12 months until data saturation occurred (eds. De Vos et al. 2011).

Consent was sought to record the collected data on a tape recorder and field notes in the local languages spoken in the Naphuno community (Sepedi, Northern Sotho and Xitsonga) and later transcribed into English for analysis.

### Data analysis

Data analysis was conducted in a qualitative manner using Braun and Clarke’s (2006:79) six steps of thematic analysis to ‘identify, analyse and report patterns (themes) within the data set and specifically for organising and describing the data set in detail’. The Braun and Clarke (2006) steps were applied as follows: firstly, becoming familiar with the data, through a detailed inspection of transcriptions. Secondly, coding was done in order to analyse and make sense of the data. Thirdly, codes were grouped into categories, and categories were consequently grouped into themes and sub-themes. The last step involved the interpretation of themes and sub-themes in terms of the research questions and objectives, and verified with the literature (Braun & Clarke 2006).

To ensure rigour and trustworthiness of the study, Guba’s model as described by Krefting (1991) was applied via three criteria: credibility, transferability and dependability. This model also involved the researcher returning to the community to confirm the findings with the participants, using the method of member checking.

### Ethical consideration

According to the ethical requirements of the study prescribed by the University of Johannesburg, Faculty of Humanities, Department of Social Work, procedures were set out for obtaining consent and maintaining confidentiality prior to facilitating this research study (ethical clearance number: 909605905).

## Results and discussion

The participants’ responses contributed to generative themes, which concluded that community members experience problems with natural hazards as a social issue, and that they needed to do community planning and intervention. Planning is, therefore, essential for ‘providing a structure to understand the factors that drive the need for an intervention’ (Weyers 2011:336). Swanepoel and De Beer (2006:37) confirmed that ‘without a need or perception of a need, community development cannot take place’.

Four themes emerged from the data and provided a comprehensive picture of the problems and needs of people who experienced natural hazards. After the discussion of the four themes, on solutions and opportunities are provided on how the problem of natural hazards could be addressed at the community level.

### Theme 1: The problems caused by natural hazards

During data analysis, the problems caused by natural hazards emerged as the first theme. Most of the participants expressed views concerning the problems caused by natural hazards. The participants revealed that natural hazards caused loss of property, material possessions, sustainable livelihood initiatives, such as subsistence farming, and bodily injuries. The following excerpts represent participants’ views in this regard:

‘Natural disasters are common in Naphuno and they destroy our household properties.’ (Participant 1, Community member from household affected by natural disasters, 17 August 2016)

‘The natural disasters mostly affect household’s properties such as furniture and electrical appliances.’ (Participant 9, experienced flooding as the house built with mud has collapsed, 15 June 2016)

‘Our food and groceries get wet when it is raining, and, thus, we lack food after heavy rains when our houses are flooded.’ (Participant 12, experienced flooding and the house built with mud was demolished, 17 August 2016)

The findings revealed the effects of natural hazards and the losses experienced by the survivors (research participants). In addition to the loss of property, such as houses, furniture and equipment, the losses suffered by survivors of natural hazards included crops and livestock, physical injuries and even death. The following comments confirm that participants had experienced these losses:

‘The heavy rain also affects our crops and sometimes destroys our livestock.’ (Group Discussion)

‘The thunderstorm blew away our roof and our houses: it specifically affects the corrugated iron roof – also known as “masenke” in Sepedi.’ (Group Discussion)

‘Our houses collapsed during natural disasters caused by heavy rain and windstorms.’ (Group Discussion).

‘The lightning does not only destroy property; it also strikes people and kills them.’ (Participant 15, lost a family member because of lightning, 15 June 2016)

‘Mostly, the disaster strikes during the night when everyone is asleep: this hinders us from receiving emergency assistance urgently as everyone is asleep.’ (Participant 17, experienced flooding as the house built with mud has collapsed; 15 June 2016)

Most participants experienced losses during natural hazards. The problems caused by natural hazards, as indicated above, are consistent with the findings of the International Federation of Red Cross and Red Crescent Societies (IFRC) (n.d.), namely, that South Africa is dominated by localised incidents of natural hazards, such as wildfires, seasonal flooding, hail and wind. Some of the problems participants mentioned were visible, such as the loss of household property, and some were not visible, such as their experiences of the loss and trauma, which emerged as a sub-theme relating to feelings of distress and hopelessness.

#### Sub-theme 1: Feelings of distress and hopelessness

Feelings of distress and hopelessness emerged as a sub-theme from the views expressed by the participants concerning the problems caused by natural hazards. The following concerns were expressed:

‘The roof of my house was blown away by the heavy windstorm. I am unable to put a roof on my house again after it was demolished.’ (Participant 10, house roof was blown away by thunderstorm, 17 August 2015)

‘We have lost our dignity and pride because of the problem of natural disasters.’ (Group Discussion).

‘We felt humiliated after we lost our belongings.’ (Group Discussion)

‘I get hurt more when people think there is something wrong when my house was blown away by wind-storm.’ (Participant 11, house roof was blown away by thunderstorm)

‘I hate it when everyone is talking about me … and feeling sorry for me.’ (Participant 17, experienced flooding as the house built with mud has collapsed, 17 August 2015)

‘I don’t like it when people feel sorry for me.’ (Participant 13, family members sustained injuries when the house collapsed on them, 17 August 2015)

‘It took years to build our houses and the natural disaster destroyed it in a flash.’ (Group Discussion)

‘I feel hopeless. How will I recover again?’ (Participant 15, lost a family member because of lightning, interviewed 15 June 2016)

The findings revealed emotional responses from participants that left them in a state of distress, hopelessness, self-pity and setback. The findings on feelings of distress and theme of hopelessness were confirmed in previous research by Dominelli (2012) and Norris et al. (2002), which reported that people who are affected by natural hazards often do not only loose property but also experience severe, lasting psychosocial problems. Similarly, Adams et al. (2002) and Freedy and Hobfoll (2013) confirmed the finding that people suffer when they have experienced significant loss, such as widespread damage to property and serious, ongoing economic difficulties caused by natural hazards. Furthermore, during analysis of the transcripts of the interviews and FGDs, it was found that the participants’ voices on audio recordings were almost inaudible when they discussed what had transpired during natural hazards. This confirmed that the research participants were sad and experienced feelings of loss as a result of the various types of natural hazards experienced by their community.

It was observed that participants who expressed feelings of sadness and hopelessness required further counselling. Thus, three research participants were referred to local social workers for counselling services, which had been arranged beforehand, during the planning of the data collection process, with social workers in the local community. The negative experiences and views of the research participants resulting from problems caused by natural hazards prompted the researcher to question how the research participants cope with the effects of natural hazards.

### Theme 2: Dissatisfaction with the disaster interventions available to Naphuno community

Data analysis of the transcripts of the FGDs revealed their dissatisfaction with the disaster intervention strategies available in their community after a natural hazard. Initially, the participants indicated the type of disaster intervention received from government by expressing the following sentiments:

‘After our houses collapsed because of heavy rain, we then notified the ward councillor who reported the disaster to the disaster management in Greater Tzaneen Municipality.’ (FGD, community members affected by natural disasters, 15 October 2016)

‘The social workers visited our homes to assess the situation after the disaster.’ (FGD, community members 1-12, 15 October 2016)

‘They take very long to help us. Since the government officials came and visited our place to see the damage caused by the disaster, they never came back to assist us.’ (Group Discussion)

‘We are not involved in community disaster interventions. The people who are supposed to assist us do not ask us what we need. They think we only need food parcels.’ (Group Discussion)

The findings of the study confirmed that social workers visit the participants to assess the impact of a natural hazard. The social workers’ home visits to the participants are characterised by screening and assessment to determine the possibility of an SRD grant. The findings confirmed the current disaster interventions’ focus on providing material assistance and reconstructing properties damaged by the natural hazard. Although government focused primarily on the physical recovery of communities, community members had negative experiences of this. The findings are furthermore supported by Huang and Wong (2013), who reported that disaster management and interventions, especially intervention by government, focused mostly on physical recovery, such as rebuilding infrastructure and house reconstruction. The findings confirmed that government plays a major role in disaster management. This is consistent with the findings of Edwards-Winslow (2002) and Quarantelli (1987), namely that governments provide a traditional model of natural hazard management through their departments and organisations – they are the only, best and most reliable responders in times of natural hazard.

Furthermore, the community intervention following disasters within the Naphuno community was identified as an intervention requirement for properly intervening in natural hazards. The findings also revealed that in the future, community disaster interventions should include the people’s own skills which they consider as community assets, which could be contributed after a community had experienced a natural hazard. The community acknowledged that although they had lost material possessions, they still possessed inherent skills and assets that can be utilised to rebuild their life following a disaster. For clarification, the gifts of the ‘head’ are things that a person knows something about and would enjoy sharing with others; gifts of the ‘hands’ are manual skills and gifts of the ‘heart’ are things that a person cares about (Kretzmann & McKnight 1977). The skills which emerged from the community members included skills, such as community-building, communication, enterprise, teaching, artistic skills, etc. (Mathie & Cuningham 2009). Table 2 presents a summary of the many skills provided by the community members in the study which they could focus on in rebuilding the community.

**TABLE 2:** Community skills and assets of the affected community members that can be utilised in disaster management.

Skills of head	Skills of hand	Skills of heart
**Conflict mediation: the ability to resolve conflicts.** **Advising: which is related to the wisdom that they possess, through which they are able to provide guidance to others.** **Counsel**l**ing: the ability to support people emotionally.** **Computer knowledge: the ability to operate a computer.** **Memorising: the ability to recall past memories vividly.** **Creative thinking: the capacity to think new ideas to improve things or the community.** **Reading: the ability to understand something that is written.** **Time reckoning: the ability to understand time and seasons.** **Imagining: being able to picture things before they happen.** **Planning: having the ability to prepare beforehand for things.** **Counting: relating to counting numbers from their head.** **Organising** **Public speaking: being able to communicate to/with a large group of people.**	Weather forecasting: the ability to predict the weather utilising indigenous knowledge. Basketry: being able to weave baskets. Plant production: to be able to produce plants, more with respect to farming. Pottery: making vases from clay. Textile making: the ability of sewing clothes or designing fabrics. Metallurgy-making: the ability to make things from iron and steel. Wood carving: the ability to make wooden sculptures. Cutting wood. Building houses. Typing. Writing. Sewing: the ability to sew clothes. Knitting: the ability to knit using wool. Touching: being able to touch and feel using hands. Driving: the ability to drive a car/vehicle. Ploughing. Painting: of houses using paint. Making art: the ability to draw. Gardening. Brick-making. Bricklaying Carpentry: being able to do woodwork, such as building of cupboards. Sweeping of floors. Cleaning: cleaning of a house. Weaving: the weaving of baskets.	Kind-heartedness. Loving: being devoted towards the development of the community. Having a sense of humour: being able to make people laugh even when they are in distress. Conflict resolution: the ability to resolve conflicts. Peacemaking: someone who does not like conflict and always ensures that there is peace between people. Willingness to collaborate: having a heart that is not selfish, but able to work with others. Caring for children: able to take care of small children.

*Source*: Focus group discussion and interview data

The findings regarding community skills and assets can be utilised in disaster interventions. The findings are consonant with the declaration by Rahmato (1991) that indigenous disaster survival strategies should involve adopting emergency resource management measures, using natural resources effectively, divesting savings and disposing of assets and making greater and more efficient use of the market system.

### Theme 3: Management of natural hazards in the future

The issue of how to manage natural hazards in the future emerged as a theme. The findings indicate that participants should observe warning signs to mitigate the impacts of natural hazards. In corroboration, the participants expressed the following views:

‘In the future, disaster intervention partnerships need to be forged with community members.’ (Group Discussion)

‘The extreme weather events that we are currently experiencing act as a warning sign for bad things that are about to happen … In this case, we should always be prepared for any natural disasters.’ (Group Discussion)

‘We are always prepared for any natural disasters by observing early warning signs, such as weather forecasting. However, due to climate change, it is very difficult to prepare for the weather these days.’ (Group Discussion)

‘We should develop emergency warning signs that our forefathers used and our local knowledge to mitigate the impacts of natural disasters.’ (Group Discussion)

There are many solutions identified in the study with the participants, such as forging partnerships with participants, warning signs predicting bad things to happen and preparation through early warning signs, and emergency warning signs should be utilised to proactively respond to impending disasters. In addition, most of the participants reported that the early and emergency disaster warnings relying on traditional belief systems of weather bulletins should be developed and strengthened. Moreover, this theme’s findings are in agreement with Gwimbi’s (2009:75) recommendation that early warning information and advice should be provided to households on how to respond to potential flood risk, as this becomes ‘one important resilient strategy to reduce risks to people and property’.

### Theme 4: Community solutions for disaster management

Community solutions for disaster management and dealing with natural hazards emerged as a fourth theme, comprising sub-themes such as collaboration with government, preparedness and after-care services.

#### Sub-theme 1: Collaboration with government to manage disasters

The participants acknowledged that collaboration with government is the first solution recommended for any disaster intervention, but emphasised that they should be involved with government to manage the natural hazards. To elaborate, the following sentiments were expressed:

‘It is everyone’s responsibility to solve the problem of natural disasters, including the government.’ (FGD, community members 1-12, 15 October 2016)

‘We should work together with government to manage disasters.’ (FGD, participant’s 9-20, 15 October 2016)

However, the participants further expressed the following sentiments:

‘A community collaboration should be integrated to guide the future disaster interventions.’ (Group Discussion)

The above findings imply that natural hazards should be addressed at a community level. The participants recommended that in order to address the problems caused by disasters, they need to be involved in decision-making. The participants indicated that their experience of disasters made them conscious of and helped them to recognise their disaster-related problems and how to solve them. However, they indicated that they were not involved in government decision-making once a disaster had occurred. The findings expressed by participants concurred with Strydom (2011) and Vollman et al. (2004), who recommended that, in alignment with PAR and community development principles, community members should be actively involved in decision-making after natural hazards.

#### Sub-theme 2: Lack of disaster preparedness and after-care services

The second solution, or opportunity identified, was about the lack of preparedness and after-care services. An example was cited regarding an incident in which a participant and members of his household were injured when their house collapsed on them. During the FGD, it was elaborated that after discharge from hospital, no intervention or social support was provided to them. The participants expressed the following sentiments:

‘I am not satisfied with the type of the intervention I have received. I am still struggling to put a roof over my head since the disaster. We still sleep with my children in a house where the temporary roof is covered by the tent provided by the municipality when we experienced a disaster last year.’ (Participant 12, experienced flooding and the house built with mud was demolished, 17 August 2016)

‘The people affected by a natural disaster, if they are admitted to a hospital because of injuries sustained during a natural disaster; when they are discharged from hospital, an after-care service in terms of support and counselling should be provided.’ (Group Discussion)

The participants further expressed the following sentiments:

‘We should be able to utilise our own assets to cope by being there and supporting each other as we do when there is a funeral or “mokete” [wedding celebration or party]. In our community, we also support each other through “sebatakgomo”, (stokvel) which aids when there is a disaster or a celebration.’ (Group Discussion)

Community members indicated their experiences of loss (property and household belongings) following extreme natural hazards, and people were injured and had to seek medical attention. The findings further revealed the greatest ‘need’ for support services beyond material assistance, such as after-care services, to contribute to the management of natural hazards in their own community. This implied that they should not be isolated clients who are only dependent on social services but should be provided with emotional support such as counselling services. Participation could involve developing preparedness and intervention plans for the community, involving people affected by natural hazards. Furthermore, an after-care service in terms of support and counselling should be provided. The participants’ views mentioned above are confirmed by Huang and Wong (2013) as well as Tanner and Mitchell (2008) that social recovery services, such as rebuilding social relationships or social functions, was rarely mentioned in government documents.

## Conclusion and recommendations

The analysis of the problems experienced in Naphuno highlighted the gaps in the general needs-based approach utilised in the management of disasters and emphasised the desired approach in disaster management, namely, the ABCD approach, based on assets. The problem analysis was problem-focused because of the nature of disasters and was conducted to support the community to gain an in-depth understanding of the problems resulting from natural hazards. The researchers acknowledge that the focus on needs served as a limitation of the overall analysis, as this study was not focused on the needs-based approach. Nevertheless, the focus on needs was necessary and had to be done to involve the community members of Naphuno in the analysis of their needs and problems. The findings presented an analysis of concerns expressed by community members with respect to natural hazards. The needs identification was done with the involvement and cooperation of the community members affected by natural hazards. Doing so was essential for determining the needs of the community. The collaborative way in which the analysis of problems of Naphuno community was done revealed how community members think their disaster planning, interventions and preparedness should be addressed. The findings of the FGD exposed an integrated community disaster intervention plan, which was devised systematically by the research participants in order to identify intervention plans that could be integrated in disaster intervention in communities affected by a natural hazards. The plan was based on their views about the problems caused by natural hazards, available disaster interventions and what should be done once people had experienced natural hazards. The main concern cited was the problem of being affected by natural hazards, and that the community members of Naphuno were not involved in the intervention process and decision-making about interventions.

The findings concluded that government should involve communities in managing natural hazards. African countries tend to expect assistance in terms of natural hazards from outside and primarily in relation to the improvement of infrastructure, health and livelihoods. The findings of this research confirmed that the community members felt disempowered to manage natural hazards on their own, and as a result, they are dependent on external sources for help when a natural hazard strikes. These challenges require increased international cooperation and the provision of adequate support to African countries to allow for the implementation of the present Sendai Framework for Disaster Risk Reduction (2015:24). The generative themes that arose from the responses in the group discussions lead to the conclusion that community members experienced problems related to natural hazards as a social issue, which required community intervention. Consequently, community members acknowledged the need for guidance and support in developing community disaster plans and guidelines for rural communities affected by natural hazards.

In analysing the community problems related to natural hazards, the community members of Naphuno established that a participatory community disaster plan should be a community-driven process, should value and appreciate the contributions of all the participants and encourage community leadership and participation. Furthermore, community development approaches, such as ABCD, should be properly integrated and implemented in the planning and development of solutions for natural hazard management endeavours for the community. Mathie and Cunningham (2009:8) affirmed that the ABCD approach can assist community members ‘to move from the position of clients to be active citizens and that communities should drive and change the course of their own development’. Therefore, it has been agreed that both experts of natural hazard management and community members should be involved in the management of natural hazards. Community members should be empowered to manage disasters on a community level and the inherent strengths of the community should be recognised.

This study has raised consciousness about the way community members could possibly contribute to rebuilding their communities by incorporating asset-based approaches after experiencing natural hazards. The findings established that proper community strategies are needed to mitigate the problems caused by natural hazards. These strategies should incorporate all stakeholders; thus, after analysing the concerns of the community in a participatory manner, it is recommended that an action committee or an enquiry group should be established to plan for the effective management of a community disaster plan – one which is facilitated by community social workers. With sufficient knowledge and support on the management of natural hazards, the community’s involvement and participation in decision-making would be paramount. An ABCD approach is pivotal for the management of community disasters in collaboration with other stakeholders, significantly, combining community problems and assets to mobilise community members’ assets to build a resilient community through ABCD approaches.
